# The Role of RNA Polymerase II Contiguity and Long-Range Interactions in the Regulation of Gene Expression in Human Pluripotent Stem Cells

**DOI:** 10.1155/2019/1375807

**Published:** 2019-02-03

**Authors:** Livia Eiselleova, Viktor Lukjanov, Simon Farkas, David Svoboda, Karel Stepka, Irena Koutna

**Affiliations:** Centre for Biomedical Image Analysis, Faculty of Informatics, Masaryk University, Botanicka 68a, Brno 60200, Czech Republic

## Abstract

The eukaryotic nucleus is a highly complex structure that carries out multiple functions primarily needed for gene expression, and among them, transcription seems to be the most fundamental. Diverse approaches have demonstrated that transcription takes place at discrete sites known as transcription factories, wherein RNA polymerase II (RNAP II) is attached to the factory and immobilized while transcribing DNA. It has been proposed that transcription factories promote chromatin loop formation, creating long-range interactions in which relatively distant genes can be transcribed simultaneously. In this study, we examined long-range interactions between the *POU5F1* gene and genes previously identified as being *POU5F1* enhancer-interacting, namely, *CDYL*, *TLE2*, *RARG*, and *MSX1* (all involved in transcriptional regulation), in human pluripotent stem cells (hPSCs) and their early differentiated counterparts. As a control gene, *RUNX1* was used, which is expressed during hematopoietic differentiation and not associated with pluripotency. To reveal how these long-range interactions between *POU5F1* and the selected genes change with the onset of differentiation and upon RNAP II inhibition, we performed three-dimensional fluorescence in situ hybridization (3D-FISH) followed by computational simulation analysis. Our analysis showed that the numbers of long-range interactions between specific genes decrease during differentiation, suggesting that the transcription of monitored genes is associated with pluripotency. In addition, we showed that upon inhibition of RNAP II, long-range associations do not disintegrate and remain constant. We also analyzed the distance distributions of these genes in the context of their positions in the nucleus and revealed that they tend to have similar patterns resembling normal distribution. Furthermore, we compared data created *in vitro* and in silico to assess the biological relevance of our results.

## 1. Introduction

Human pluripotent stem cells (hPSCs), including both human embryonic stem cells (hESCs) [1] and human induced pluripotent stem cells (hiPSCs) [2], are capable of self-renewal and differentiation into all germ layers. Although extensive attention has been dedicated to uncovering their underlying characteristics, the genome spatial organization and chromatin dynamics during the switch from the pluripotent to the differentiated state remain to be elucidated. Nevertheless, understanding these processes appears crucial for future clinical applications of hPSCs. The situation in pluripotent nuclei seems to be far more complex than that in differentiated nuclei, and pluripotent nuclei have unique epigenetic features [3–7]. One of the central mechanisms responsible for lineage specification and cell fate determination is transcriptional regulation [8], suggesting that the assembly of pluripotency genes in specialized structures known as transcription factories (TFs) is required for the maintenance of pluripotency.

It has been shown that transcriptionally active genes associate with TFs, described as discrete nuclear sites of nascent RNA molecules wherein transcription components are concentrated [9–11]. This strategy to transcribe several genes simultaneously involving the same TF seems to be conserved and efficient since DNA replication and nucleolus transcription machinery share the same patterns [12, 13]. Active transcription machinery involves the active phosphorylated form of RNA polymerase II (RNAP II), transcription factors, and other cofactors recruited by enhancer elements. Enhancers are DNA elements that are brought into proximity with promoters of transcribed genes, promoting chromatin loop formation. As previously shown, enhancers not only stimulate transcription from the nearest promoter but also modulate the transcription of distant promoters or even promoters on different chromosomes [14].

Chromatin loops are responsible for long-range interactions defined as crosstalk between enhancer elements and distally positioned genes, thus regulating the transcription of relatively distant genes [15–18]. As has been demonstrated, the same TF can be used for the transcription of several genes simultaneously [19]. This observation was fueled by other research showing that distal genes are dynamically organized and colocalize to the same TF at high frequencies by migrating to preassembled transcription sites [20]. During early embryogenesis, enhancer elements marked with different chromatin signatures either activate or suppress the transcription of nearby genes [21], suggesting that lineage specification of hPSCs leads to an extensive reorganization of nuclear architecture [22]. As has recently been shown, chromatin interactions, both within and between chromatin domains, change in a remarkable manner, modifying up to 36% of active and inactive chromosomal regions throughout the genome [5].

The transcription of active genes in TF is carried out by RNAP II. Transcription itself is a multistep process, starting with the inactive unphosphorylated form of RNAP II binding to DNA. For transcription initiation, RNAP II phosphorylation at the Ser5 and Ser7 positions of the C-terminal domain (CTD) by cyclin-dependent kinase 7 (CDK7) is required. Elongation factor (P-TEFb) containing the CDK9 kinase subunit is mandatory to progress into the next stage of transcription; thus, inhibitors of the CDK9 kinase result in the inhibition of transcription elongation. Today, many RNAP inhibitors that target different stages of the transcription process are available [23]. Many compounds that inhibit transcription have useful pharmacological properties, namely, several CDK9 inhibitors. Flavopiridol has been described as a transcription inhibitor, preventing entry into the transcription elongation phase by inhibiting CDK9 [24, 25]. Due to its unique mechanism of action, flavopiridol seems to be the most promising transcription inhibitor, and several clinical trials using this powerful drug in chemotherapy have been reported [26, 27].

Recently, long-range interactions and their role in the pluripotency and differentiation of hPSCs have been evaluated [18]. It has been shown that the well-known pluripotency gatekeeper Oct4 (encoded by the human gene *POU5F1*) has a huge impact not only on pluripotency maintenance but also on differentiation by directing the cell fate toward a primitive endoderm and mesoderm [28]. Using circular chromosomal conformation capture with high-throughput sequencing (4C-seq), chromosomal regions that colocalize frequently with the Oct4 locus in PSCs were identified [29–31]. These interactions are established prior to transcriptional activation, mediating DNA looping at the Oct4 enhancer.

In the present study, we focused on the pluripotency master gene *POU5F1* that encodes the transcription factor Oct4. We performed three-dimensional fluorescence in situ hybridization (3D-FISH) analysis to reveal long-range interactions between *POU5F1* and the genes *CDYL*, *TLE2*, *RARG*, and *MSX1*, previously identified as possible interacting partners [30]. As a control, a gene not associated with pluripotency, *RUNX1*, was chosen. This gene is believed to play a role in hematopoietic differentiation [31]. We sought to know whether and how these possible interactions fluctuate upon differentiation or while pausing transcription using elongation inhibitors of RNAP II. As a next step, we were interested in the distance and spatial distribution of signals throughout pluripotent and differentiated nuclei. Finally, we combined these biological data with computational analysis by creating artificial nuclei with randomly loaded signals to test the randomness of the observed interchromosome associations. Our goal was to reveal whether these gene combinations could be members of the same TFs using long-range interaction analysis.

## 2. Materials and Methods

### 2.1. Cell Culture

The human ESCs employed in this study were derived from blastocyst-stage embryos obtained with the informed consent from the donors. A well-characterized hESC line [32], CCTL14 (Center of Cell Therapy Line 14), was used in passages 29-41. For human iPSCs, the well-characterized line ID CBIA-19, derived in our laboratory from human umbilical vein endothelial cells using genome nonintegrating vectors [33], was used from passages 34 to 41. All hPSCs were grown on a Matrigel™ matrix (Thermo Fisher Scientific, Waltham, Massachusetts, USA) in mTESR™1 (Stemcell Technologies, Vancouver, Canada). To induce differentiation, hPSCs were cultured in DMEM/F12 (Life Technologies, Carlsbad, California, USA) containing the GSK3*β* inhibitor CHIR 99021 (4 *μ*M) (Sigma-Aldrich, St. Louis, Missouri, USA) for three consecutive days.

### 2.2. BAC DNA Isolation and Precipitation

*Escherichia coli* carrying plasmids with sequences CTD-2534O14, RP11-624J6, RP11-346E12, RP11-268O21, RP11-1006L1, and RP11-620A17 were chosen from http://www.ncbi.nlm.nih.gov and acquired from Life Technologies BAC library (see Supplementary Figure 1). Plasmid DNA isolation from *E. coli* was carried out using a Qiagen® Large-Construct Kit (Qiagen, Hilden, Germany) that is specifically used for the isolation of genomic DNA-free BAC DNA according to the manufacturer's instructions. The isolated BAC DNA was resuspended in TE buffer and quantified with the NanoDrop 2000 spectrophotometer (Thermo Fisher Scientific). For BAC DNA precipitation, 2 *μ*g of DNA was mixed with 6 *μ*l of Cot DNA and 1.4 *μ*l of DNA (MB grade, Sigma-Aldrich). The mixture was incubated at room temperature for 5 min, followed by the addition of 1.5 *μ*l of 3 M acetic acid and 22 *μ*l of cold 70% ethanol and incubation at -80°C for 30 min. After incubation, the samples were centrifuged at 12000 rpm at 4°C for 30 min, the supernatant was removed, and the pellet was allowed to air-dry. Precipitated BAC DNA was resuspended in 3 *μ*l of PCR water and stored at -80°C.

### 2.3. NICK Translation and DNA Probe Preparation

NICK translation for BAC DNA was carried out using a mixture of DNA probes labeled with digoxigenin-11-dUTP DIG-NICK Translation Mix and biotin-16-dUTP Biotin-Nick Translation Mix (both purchased from Sigma-Aldrich) according to the manufacturer's instructions. Briefly, 1 *μ*g of template DNA was mixed with 4 *μ*l of NICK translation mix and double-distilled water and incubated at 15°C for 90 min. The reaction was stopped by adding 1 *μ*l of 0.5 M EDTA and heating at 65°C for 10 min. Prior to adding EDTA, the proper length of fragments was verified with 3 *μ*l of NICK-translated DNA on a 1% agarose gel. DNA probes were aliquoted and stored at -80°C.

### 2.4. Three-Dimensional Fluorescence In Situ Hybridization (3D-FISH)

To perform 3D-FISH, hPSCs and their differentiated counterparts were cultured on coverslips, fixed with 4% paraformaldehyde (Sigma-Aldrich) for 10 min at room temperature (RT), washed in PBS (3× for 5 min), permeabilized with 0.5% Triton X-100 in PBS for 30 min, equilibrated in 2× SSC, and treated with RNase A (10 *μ*g/ml, Qiagen) at 37°C for 1 hour. After washing the cells again in 2× SSC and PBS, they were incubated in 0.1 N HCl, permeabilized with 0.2% saponin/PBS and 100 mM Tris-HCl, and briefly rinsed in 2× SSC. Cellular DNA was treated with a hybridization mixture (70% formamide/2× SSC) at 75°C for 5-6 min and dried in an ethanol series. After DNA probe denaturation (75°C for 7 min) and reannealing (37°C for 30 min), the probes were mixed with Hybrisol VII (Qbiogene, Carlsbad, California, USA), applied to cells, sealed under coverslips with rubber cement, and allowed to hybridize overnight. After hybridization, the slides were washed in 50% formamide and 2× SSC at 45°C and then incubated with anti-digoxigenin-rhodamine Fab fragments and avidin fluorescein conjugate (both purchased from Sigma-Aldrich) for 1 h at 37°C. After rinsing the cells with 2× SSC/0.1% Tween, the coverslips were mounted in DAPI-containing Mowiol (Sigma-Aldrich).

### 2.5. Immunocytochemistry

Cells were fixed with 4% paraformaldehyde (20 min, RT), permeabilized with 0.1% Triton-X100 in PBS (20 min, RT), and incubated with primary antibodies at 4°C overnight. The primary antibodies included goat polyclonal anti-Oct3/4 (Santa Cruz Biotechnology, Dallas, Texas, USA), rabbit monoclonal anti-Nanog (Cell Signaling Technology, Danvers, Massachusetts, USA), and rabbit anti-RNAP II Ser2 (Abcam, Cambridge, UK). The next day, incubations with secondary antibodies conjugated to Alexa Fluor 488 or Alexa Fluor 594 (Cell Signaling Technology, Leiden, Netherlands) were carried out at RT for 1 hour. Coverslips were mounted in DAPI-containing Mowiol (Sigma-Aldrich). Microscopic analysis was performed using a Zeiss Axiovert 200M system (Carl Zeiss, Oberkochen, Germany).

### 2.6. Flow Cytometry

To determine the expression of cell surface antigens, we used flow cytometry. Briefly, cells were harvested using trypsin/EDTA, resuspended in PBS/0.5% BSA/2 mM EDTA, and incubated with fluorochrome-conjugated antibodies at 4°C for 10 min. The antibodies included SSEA-4 (phycoerythrin-conjugated antibody: PE; R&D Systems, Minneapolis, Minnesota, USA) and Tra-1-60 and Tra-1-81 (both PE, Miltenyi Biotec, Bergisch Gladbach, Germany). For an isotope control, a mouse IgG3 antibody (PE, R&D Systems) was used. Samples were measured with a BD FACSCanto II flow cytometer (Becton Dickinson, Franklin Lakes, New Jersey, USA). For data analysis, Flowing Software (Cell Imaging Core, Turku, Finland) was used.

### 2.7. Confocal Microscopy and Image Analysis

The specimen slides were imaged using a Zeiss Axiovert 200M system (Carl Zeiss) confocal microscope with a 40x oil objective lens. The imaged field of view (FOV) was acquired at a resolution of 1024 × 1024 pixels. A stack of 60 digital image slices was acquired with a 0.2 *μ*m step interval. Thus, these images fully covered the 3D volume of the specimen, making the *z* resolution of the image slice sufficient for detecting two FISH-probed signal spots. The acquired image slices were saved in the red/green/blue (RGB) format. The 3D interphase nucleus was assembled from the volume of interest of the RGB image stack using the open-source software Acquiarium (https://cbia.fi.muni.cz/software/acquiarium.html).

### 2.8. Simulation of Nuclei In Silico

The simulated images were generated in a virtual microscope [34] using a parametric-based approach [35]. In real cells, only the interior of nuclei are fluorescently stained. Therefore, we modeled only the nuclei, ignoring the cytoplasm. Each cell was modeled in two channels. The first channel corresponded to DAPI, showing the nucleus mask, its structure, and the nucleoli. The shape was defined as a moderately deformed ellipsoid, with the chromatin imitated using a procedural texture [36]. Nucleoli were created as gaps in the texture, randomly placed within the nucleus. The second channel corresponded to Oct4 with the selected genes. Like nucleoli, the genes were randomly positioned within the nucleus. Finally, these two channels were merged together to imitate the pseudocolor RGB image. To fill the whole microscopic slide with more cells, we used the procedure repeatedly while controlling the distribution of cells across the slide [37].

## 3. Results

### 3.1. Analysis of Long-Range Interactions in hPSCs between *POU5F1* and the Selected Genes Reveals the Highest Numbers with the *CDYL* Gene and the Lowest Numbers with the *RUNX1* Gene Loci

**Figure 1:**
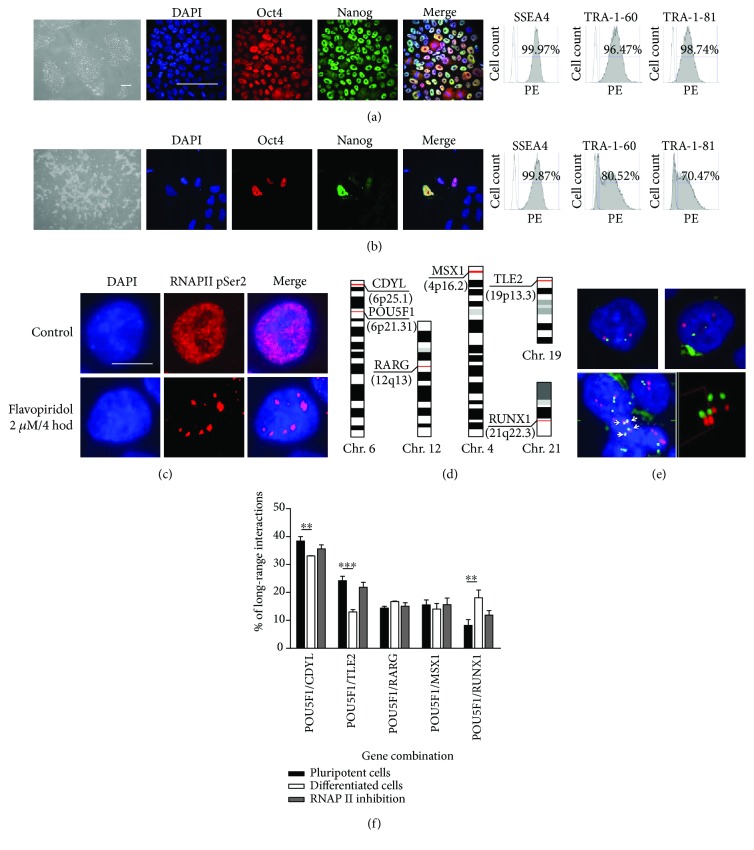
Long-range interactions between *POU5F1* and the selected gene loci in human pluripotent stem cells (hPSCs) and early differentiated cells and upon RNAP II inhibition. (a) Human PSCs display normal undifferentiated morphology and maintain pluripotent marker expression. Right panel: immunocytochemistry for Oct4 (red) and Nanog (green). Left panel: flow cytometry of hPSCs positive for SSEA4, TRA-1-60, and TRA-1-81 indicated in %. Scale bar: 100 *μ*m. (b) Change of medium composition causes rapid differentiation leading to alteration of hPSC morphology and loss of pluripotent markers Oct4 and Nanog while only slow reduction in expression of surface markers SSEA4, TRA-1-60, and TRA-1-81 was observed. (c) Immunocytochemistry of the active phosphorylated form of RNAP II shows that transcription occurs in discrete loci within the nucleus possibly forming transcription factories (upper panel). After RNAP II inhibition, transcription remains only in isolated regions within the nuclei (bottom panel). Scale bar: 10 *μ*m. (d) Arrangement of the selected genes on chromosomes. (e) Two different genes located on the same chromosome (*POU5F1* in green and *CDYL* in red, both on chromosome 6) show close proximity, although they can be separated by long distances (upper panel). While signals produced by FISH in a 2D image show close proximity and possible colocalization (upper panel), 3D-FISH visualization reveals distance between them (bottom panel). (f) Long-range interactions between *POU5F1* and the selected gene loci in hPSCs and their early differentiated counterparts and upon RNAP II inhibition. As a criterion for long-range interaction, distance ≤ 2 *μ*m between two FISH signals was chosen. Representative images showing the human iPSC line ID CBIA-19 in passage 35 are shown. Cell nuclei were counterstained with DAPI (blue). For 3D-FISH, at least 500 nuclei for each gene combination were counted (*n* = 2). In charts, columns show means and error bars show SEM. Student's *t*-test, ^∗∗∗^ *p* < 0.001, ^∗∗^ *p* < 0.01, and ^∗^ *p* < 0.05.

To reveal long-range interactions between *POU5F1* and the selected genes in TFs formed in hPSCs, we used both hiPSCs and hESCs with typical undifferentiated morphology and expression of pluripotent markers (Figure 1(a)). To examine long-range interactions in early differentiated cells, we used media supplemented with CHIR 99021—a potent GSK3*β* inhibitor that drives hPSCs to become mesoderm [38]. Upon this differentiation, hPSCs rapidly change their morphology and lose the expression of pluripotency markers (Figure 1(b)), suggesting rearrangement of nuclear topology. To study the effect of RNAP II inhibition on long-range interactions in hPSCs, we inhibited RNAP II using an inhibitor that selectively pauses the transcription elongation phase. For this purpose, we chose flavopiridol, a well-documented inhibitor of transcription elongation. First, we used immunocytochemistry to evaluate the distribution of active RNAP II before and after inhibition. In accordance with previously published data, active RNAP II was localized in discrete regions throughout the nuclei (Figure 1(c)). Upon inhibition, transcription gradually decreased in a time-dependent manner until the cell cycle was halted, and only a few discrete clusters of transcription active sites remained (Supplementary Figure 2).

We performed 3D-FISH analysis to visualize long-range interactions between *POU5F1* and genes that have previously been identified as *POU5F1* enhancer-interacting partners [30]. These genes included *CDYL* (located on chromosome 6), *TLE2* (chromosome 19), *RARG* (chromosome 12), and *MSX1* (chromosome 4). As a control, the *RUNX1* gene (chromosome 21) was chosen, which is a transcription factor that regulates the differentiation of hematopoietic stem cells into mature blood cells [31] (Figure 1(d)). As indicated, both *POU5F1* and *CDYL* are located on chromosome 6, which should naturally increase the percentage of close colocalizations. Interestingly, although these genes are positioned on the same chromosome, they can be separated by long distances (Figure 1(f)). The advantage of 3D-FISH is that the assessment of real distances in 3D nuclei and 2D simulation may show the proximity of genes that can be overlapped (Figure 1(e), bottom panel). The fractionalization of nuclei by 60 sections in the Z plane improved the visualization and positions of genes in 3D nuclei (Supplementary Figure 3(a)). As a criterion for the existing long-range interactions, a distance lower or equal to 2 *μ*m (Supplementary Figure 3(b), in red circle) was chosen. As expected, the percentage of long-range interactions between the *POU5F1* and *CDYL* genes was elevated compared to the percentage of those between *POU5F1* and the other selected genes, reaching up to 39% (Figure 1(f)). The other examined genes were not substantially different. The percentage of long-range interactions did not exceed 24.23% for the *POU5F1*-*TLE2* combination, 14.4% for *POU5F1*-*RARG*, and 16% for *POU5F1*-*MSX1*. It is noteworthy that long-range interactions between *POU5F1* and the control gene *RUNX1* showed the lowest number of all the long-range interactions followed (8.22%), implying that this gene does not play a role in maintaining hPSC pluripotency (see Supplementary Table 1 for the results in % of all long-range interactions).

### 3.2. Analysis of Long-Range Interactions between *POU5F1* and the Selected Gene Loci in Early Differentiated Cells Shows Decreased *POU5F1-CDYL* and *POU5F1-TLE2* Interactions Compared to Those in Pluripotent Cells

Using 3D-FISH analysis, we also examined how long-range interactions between *POU5F1* and the selected genes changed during early differentiation. Compared to hPSCs (Figure 1(f)), we observed the most remarkable difference in the *POU5F1*-*CDYL* long-range interactions, where they dropped from 38.5% to 30%. We also detected a dramatic reduction in *POU5F1*-*TLE2* long-range interactions from 24% to 13%, suggesting that these genes do not play a role in differentiation. When analyzing *POU5F1*-*RUNX1* interactions, we observed a precipitous increase in long-range interactions from 8.22% to 18%, demonstrating the potential involvement of this gene in differentiation (Figure 1(f), see Supplementary Table 1 for the results in % of all long-range interactions). In addition, *POU5F1*-*RARG* and *POU5F1*-*MSX1* long-range interactions during early differentiation remained at approximately the same level (Figure 1(f), see also Supplementary Table 1).

### 3.3. Previously Formed Long-Range Interactions Remain Unaffected by RNAP II Inhibition

Next, we were interested in whether pausing RNAP II changed the formation of TFs or modified the number of long-range interactions we observed previously in control pluripotent cells. According to our hypothesis, the inhibition of transcription elongation may stabilize transcription factories while loading more RNAP II onto the promotor, and the percentage of long-range interactions should remain at the same level. To address this issue, we inhibited transcription elongation using flavopiridol and performed 3D-FISH to assess the long-range interactions between the *POU5F1* gene and its interacting partners. As a result, analysis of long-range interactions showed results similar to those from control hPSCs, suggesting that transcription factories remained stable and constant (Figure 1(f), Supplementary Table 1). The only exception was the *POU5F1*-*RUNX1* combination, where we observed a slight increase in long-range interactions from 8.2 to 11.9% (Figure 1(f), Supplementary Table 1). To conclude, we assume that the inhibition of transcription has no influence on preexisting transcription factories and once created, they remain constant.

### 3.4. Pluripotency, Differentiation, or RNAP II Inhibition Does Not Influence the Pairing of Individual Alleles

**Figure 2:**
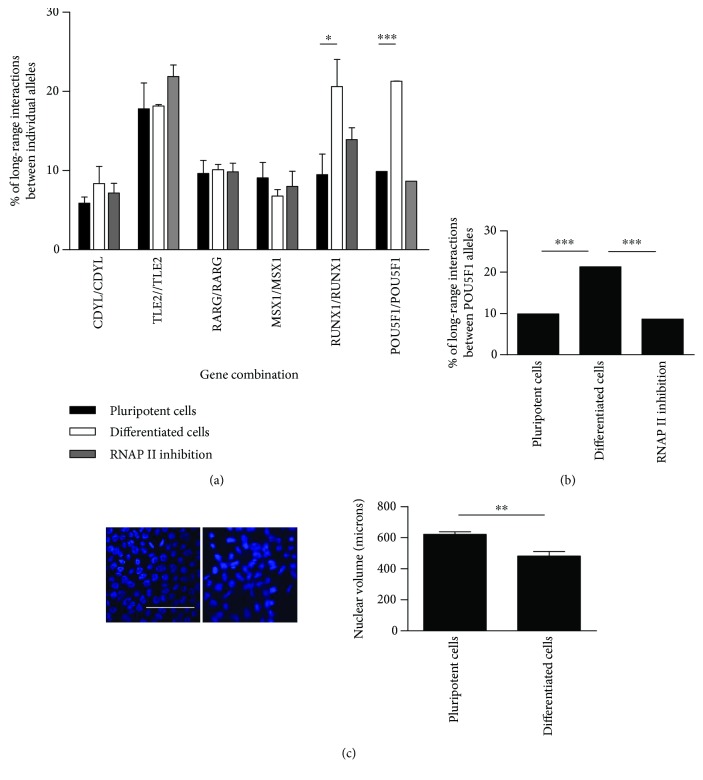
Long-range interactions between individual alleles of the selected genes and *POU5F1* in hPSCs and early differentiated cells and upon RNAP II inhibition. (a) Analysis of long-range interactions between individual alleles of the selected genes in hPSCs shows that these allelic associations do not change during differentiation and upon RNAP II inhibition. Only *RUNX1*-*RUNX1* allelic association increases from 8.22% to 18.1% with differentiation, and *POU5F1*-*POU5F1* increases from 9.9% to 21.3%. (b) Analysis of long-range interactions between individual alleles of *POU5F1* in more detail shows a two-fold increase in long-range interactions during differentiation from 9.9% to 21.3%. Upon RNAP II inhibition, long-range interactions remain stable or show a slight decrease compared to those in control undifferentiated hPSCs. (c) This increase in *POU5F1* allele pairing during differentiation cannot be explained by reduction of nuclear size and volume of early differentiated cells. Representative images showing the hiPSC line ID CBIA-19 in passage 35 are shown. Cell nuclei were counterstained with DAPI (blue). Scale bar: 10 *μ*m. For 3D-FISH, at least 500 nuclei for each gene combination were counted (*n* = 2). In the case of *POU5F1*, *n* = 10. In charts, columns show means and error bars show SEM. Student's *t*-test, ^∗∗∗^ *p* < 0.001, ^∗∗^ *p* < 0.01, and ^∗^ *p* < 0.05.

We also focused on long-range interactions between individual alleles of the selected genes to assess whether they can be transcribed in the same transcription factory. We demonstrated that these interchromosome associations do not happen since we did not observe any alterations in the long-range interaction patterns of allelic pairs of *CDYL*, *TLE2*, *RARG*, or *MSX1* during pluripotency and differentiation or upon RNAP II inhibition (Figure 2(a)). We can conclude that long-range interactions between allelic pairs of the selected genes do not change during differentiation or upon RNAP II inhibition, suggesting that allelic pairing of these genes is not critical for controlling pluripotency or differentiation. The highest number of long-range interactions was observed in individual alleles of the *TLE2* gene (up to 22%), although they barely changed during different experimental conditions. Interestingly, only *RUNX1* alleles displayed an increase in long-range interactions from 9.5% in pluripotent cells to 20.6% in their early differentiated counterparts (Figure 2(a), see Supplementary Table 2 for the results in % of all long-range interactions).

### 3.5. Long-Range Interactions between Allelic Pairs of *POU5F1* in Early Differentiated Cells Doubled Compared to Those in Pluripotent Cells, and This Rise Was Not Explainable by Morphological Changes or Nuclear Size Reduction

Interestingly, we detected a significant increase in long-range interactions between *POU5F1* allelic pairs during differentiation, and the signal proximity doubled in the nuclear space from 9.9% in pluripotent cells to 21.3% in their early differentiated counterparts (Figure 2(b)). As mentioned above, the selected genes other than *RUNX1*-*RUNX1* remained at approximately the same level. This observation can be explained by the diverse and opposite role of Oct4 in pluripotent cells; Oct4 governs pluripotency in hPSCs and their differentiation [39]. We wondered whether this remarkable long-range interaction increase is in accordance with morphological alterations of differentiating nuclei. We noticed that the nuclear morphology of hPSCs changed during differentiation and was also accompanied by a nuclear size reduction. In this context, the approximation of long-range interactions between both alleles of all genes would be a logical outcome. However, when we compared the volumes of nuclei from hPSCs and early differentiated cells, we did not observe this expected correlation (Figure 2(c)). Whereas a nuclear volume reduction of approximately 20% occurred during differentiation, long-range interactions between *POU5F1* allelic pairs increased twice, suggesting the dual role of Oct4 in differentiation. Upon RNAP II inhibition, *POU5F1*-*POU5F1* long-range interactions remained at the same level or displayed only a slight decrease compared to those in control undifferentiated hPSCs (Figure 2(b), see also Supplementary Table 2).

### 3.6. The Distance Distributions between *POU5F1* and the Selected Genes Display Similar Patterns and Do Not Change during Differentiation or upon RNAP II Inhibition

Furthermore, we were interested in the distribution of signal spatial distances created by 3D-FISH throughout the nuclei of pluripotent cells and their differentiated counterparts and upon RNAP II inhibition. To detect all signal arrangements, we separated signals produced by 3D-FISH in 14 intervals to cover not only the appearance of the selected genes in the nucleus (Supplementary Figure 3b) but also the chromosomal positions at which these genes are located. We found that the majority of signals are located in relative proximity, with many of them occupying the average distance of approximately 5 *μ*m. In addition, the patterns of signal distributions of *POU5F1* and the selected genes tend to have a similar arrangement; the majority of signals from the selected genes lie in close proximity, implying the proximity of chromosomes. We can conclude that the distance distributions of signals produced by 3D-FISH resemble their normal distribution (Supplementary Figure 4). We were also interested in the distance distributions of individual alleles of the selected genes, and again, there were no significant changes compared with the distribution of the alleles of these genes throughout the nuclei. Interestingly, we noticed a small variation when comparing pluripotent stem cells with their differentiated counterparts. Distances between alleles tended to be more closely grouped, which can be explained by a reduced size of differentiated cell nuclei (Supplementary Figure 5).

### 3.7. Comparison of Computer-Generated Image Data with Real Data Shows the Biological Relevance of Long-Range Interactions between *POU5F1* and the Selected Genes

**Figure 3:**
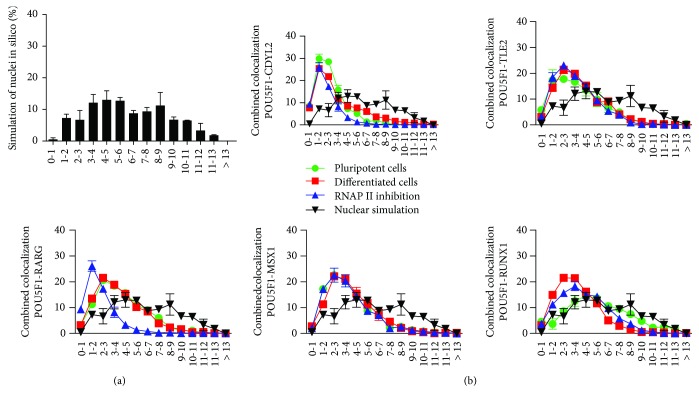
Comparison of long-range interactions and distance distribution between POU5F1 and the selected genes *in vitro* and in silico. (a) Simulation of two signals randomly positioned inside artificial nuclei shows normal distribution of distances. (b) Combination of *POU5F1* and the selected genes shows nonrandom distribution in hPSCs and their early differentiated counterparts and upon RNAP II inhibition, compared to computationally generated data.

To confirm our biological data, we compared them with randomly generated signals in artificial nuclei. We computationally constructed artificial nuclei using preexisting real data and randomly loaded signals to compare data observed *in vitro* with those created in silico. Then, we analyzed the artificial nuclei to determine long-range interactions. Randomly distributed signals showed 7.79% of the long-range interactions between signals. Similarly, the number of colocalizations increased on the opposite side of the distance spectrum, as we observed a higher number of signals at a more than 10 *μ*m distance (Figure 3(a)). When comparing the real biological data with computational analysis, we revealed differences in distribution patterns. To address the biological relevance of previously described results, we overlapped all combinations of the selected genes in pluripotent cells, their early differentiated counterparts, and cells with inhibited transcription and combined them with computationally simulated data (Figure 3(b)). For the *POU5F1*-*CDYL* combination, we observed variations from biological data. However, this effect can be attributed to the positions of these two genes on the same chromosome, something computational analysis did not take into consideration. Additionally, other genes showed slight variations from the simulated patterns. We conclude that the data presented in this study have biological resemblance that cannot be coincidental.

## 4. Discussion

Genomes are highly organized spatially within interphase nuclei. Although higher-order chromatin organization is increasingly linked to genome function and regulation of gene expression, this level of organization in hPSCs remains poorly explained. In our present study, we describe long-range interactions between the *POU5F1* gene, which codes for the well-documented pluripotency marker Oct4, and various genes previously shown to play key roles in the pluripotency maintenance of hPSCs [30, 40–42]. The long-range chromatin interactions of distant genes can thus be evidence that those genes are components of the same transcription factory being transcribed simultaneously [43, 44]. Our study addresses the question of how these long-range interactions change over time during the shift from the pluripotent state to the differentiated state and upon RNAP II inhibition. In addition, long-range interactions were followed in two physiological combinations: the distance between the *POU5F1* gene and the selected genes (i) and the distance between allelic pairs of individual genes (ii). We demonstrated that long-range interactions between two of the four selected genes decreased upon the early differentiation onset, highlighting the role of these genes in pluripotency. In addition, we showed that once created, these long-range chromosome interactions remained stable and did not disintegrate upon RNAP II inhibition.

To assess long-range interactions between the selected genes, we used 3D-FISH, a commonly used approach that allows direct visualization of the transcription and gene positions in single cells [45]. Performing 3D-FISH in pluripotent cells may be problematic since the central nuclear region is occupied mostly by open active euchromatin [6, 7, 46], and the DNA denaturation necessary for probes to attach to designed DNA sequences may cause nuclear breakdown. In addition, this approach must be time-limited to preserve the nuclear architecture. Denaturation for a prolonged period of time causes the nuclear region to collapse and the nuclei to lose their unique shape features [47]. To maintain the nucleus as intact as possible, we optimized the FISH protocol for hPSCs to help visualize the signals (see Materials and Methods).

As a criterion for long-range interaction between two FISH signals, a distance less than or equal to 2 *μ*m was chosen. Although this distance may seem too long, an open form of transcriptionally active pluripotent chromatin is a property of a pluripotent nucleus. Moreover, it has been shown that enhancer-promotor communication requires chromatin looping [14]. Loops form because two genes on the same chromosome require the same transcription machinery found in a specific TF. This requirement will attract the gene loci to the factory, thus creating a loop and forming long-range interactions [16, 48]. Nevertheless, this radius is in accordance with previous studies dedicated to interchromosomal associations [49–52].

Hi-C analysis, which reveals the genome at the megabase scale, has been used to partition the genome into two main compartments, named A and B [52], corresponding to open and closed chromatin, indicating active euchromatin and inactive heterochromatin, respectively. Another extensive study analyzing single-cell data found that while chromosome structure varies markedly from cell to cell, regions belonging to the A and B compartments always cluster together and A segregates from B, forming an outer B compartment ring, an inner A compartment ring, and an internal B compartment around the nucleoli [53], suggesting that transcriptionally active chromatin tends to occupy specific regions in the nuclei. We observed a similar tendency herein; the genes we followed during pluripotency and differentiation are at different distances and seem to cluster together, implying that the switch from pluripotency to differentiation is accompanied by remarkable morphological changes. This alteration of morphology is also accompanied by vast modifications of nuclear topology and architecture [4, 5, 54].

In this study, we showed that long-range interactions between the selected genes in pluripotent nuclei changed with differentiation. One of the genes in which this alteration is the most striking is *RUNX1*. Long-range associations with *POU5F1* gene loci increase from 8 to 18% during early differentiation, suggesting its role in this process. Indeed, RUNX1 is a member of the core-binding factor family of transcription factors and is indispensable for the establishment of definitive hematopoiesis in vertebrates. *RUNX1* is one of the most frequently mutated genes in a variety of hematological malignancies [55]. Moreover, the early differentiation we used was triggered by the withdrawal of media necessary for the maintenance of undifferentiated hPSC growth and exchange for media supplemented with CHIR 99021, a potent GSK3*β* inhibitor that drives hPSCs into becoming mesoderm [38]. We confirmed our former hypothesis that long-range interactions between *POU5F1* and *RUNX1* increase upon this type of differentiation. In addition, when spatial distributions and distances higher than 10 *μ*m were compared, we observed large shifts in *POUF51*-*RUNX1* associations to opposite nuclear regions.

For *POU5F1* and *CDYL* associations, we report a decrease in long-range interactions from 38.51 to 30% during differentiation. Interestingly, we found that being positioned on the same chromosome does not always guarantee close proximity of genes, and on the contrary, many genes are located at long distances apart, indicating transcriptionally active and open states of chromatin in pluripotent nuclei. The role of this gene in pluripotency remains unclear, although it has been implicated in chromatin remodeling and linked to X-chromosome inactivation during early embryo development. As shown in mouse embryonic stem cell lines, for its association with Xi, *CDYL* requires the process of differentiation and the presence of H3K9me2 and H3K27me3, which both become chromosomally enriched [56]. As demonstrated, Cdyl^−/−^ mouse fibroblasts can be reprogrammed into iPSCs with decreased spontaneous neuronal differentiation [57].

In addition, the *POU5F1*-*TLE2* combination follows an interesting course during the shift from pluripotency to differentiation. When analyzing *POU5F1*-*TLE2* long-range interactions, we observed a remarkable 10% drop in long-range interactions between these two genes during differentiation, suggesting a role of *TLE2* in maintaining pluripotency and undifferentiated hPSC growth. In contrast, it has been shown that *TLE2* regulates ventral telencephalon formation [58]. Among its related pathways, activation of Notch signaling has been reported to govern cell fate choices in undifferentiated hPSCs to form the progeny of all three embryonic germ layers [59]. On the other hand, inhibition of Notch signaling facilitates the differentiation of human induced pluripotent stem cells into neural stem cells [60].

With regard to *POU5F1*-*RARG* long-range interactions, our study showed no significant modifications during the onset of differentiation, which is notably interesting. In fact, it has been shown previously that RARG can favorably enrich the former transcription factor cocktail (Oct4, Sox2, Klf4, and c-Myc) used for reprogramming somatic cells into hPSCs, making this process more efficient [61]. This controversy may be explained by its ambivalent role in pluripotency and differentiation. Recent evidence suggests that in pluripotent cells, RARG interacts with genomic regions characterized by the binding of pluripotency-associated factors, while in differentiated cells, RARG-bound regions are enriched in functional Sox17-binding sites [62].

One of the most interesting outcomes of our study on interchromosomal associations is the observation of pairing between individual alleles of *POU5F1*, exhibited by approximately 10% of pluripotent nuclei and 20% of differentiated nuclei (twofold higher). As previously demonstrated, Oct4 plays a dual role in pluripotency and differentiation [39, 63, 64]. This is in accordance with a previously published study that followed Oct4 alleles during early differentiation and observed decreased Oct4 expression but elevated colocalizations at enhancer/promoter regions at the same time [52]. We conclude that *POU5F1* remains stacked in TFs during pluripotency and differentiation.

Collectively, we can summarize that long-range interactions and gene regulation are in a firm, causal relationship. Pluripotent nuclei exhibit unique features that are modified due to chromatin remodeling upon the onset of differentiation. Based on our data, we propose that upon differentiation, long-range associations between specific pluripotent genes associated with pluripotency decrease. RNAP II inhibition seems to have no impact on these ongoing long-range interactions, suggesting that the assembly of transcription factories remains constant and stable. Sophisticated high-resolution approaches analyzing the interactomes of the entire cell populations provide the same evidence.

## 5. Conclusions

In this study, we examined long-range interactions between the *POU5F1* gene and genes previously identified as being *POU5F1* enhancer-interacting, namely, *CDYL*, *TLE2*, *RARG*, and *MSX1* (all involved in transcription regulation), in human pluripotent stem cells (hPSCs) and their early differentiated counterparts using 3D-FISH followed by computational simulation analysis. We sought to elucidate how these interactions change over time and upon RNAP II inhibition. Our analysis showed that long-range interactions with the CDYL, TLE2, and RUNX1 genes change during differentiation, suggesting that transcription of the monitored genes is associated with pluripotency. In addition, we show that upon inhibition of RNAP II, long-range associations do not disintegrate and remain constant. Furthermore, we show that the pairing of individual alleles of the selected genes is affected neither by differentiation nor by RNAP II inhibition. Another interesting outcome of our study is that the number of long-range interactions between *POU5F1* individual alleles in early differentiated cells was doubled compared to that in pluripotent cells. Furthermore, distance distributions between *POU5F1* and the selected genes display a similar pattern and do not change during differentiation or upon RNAP II inhibition. As a subsequent step, we computationally constructed artificial nuclei using preexisting real data and randomly loaded signals to compare data created *in vitro* and in silico and confirm the biological relevance of our results of long-range interactions between *POU5F1* and the selected genes.

## Data Availability

Most of the data (microscopical images, results from flow cytometry, and results from 3D-FISH analysis) used to support the findings of this study are all included within the article and also within the supplementary information file. The raw data (microscopical images for 3D-FISH and 3D-FISH analysis) used to support the findings of this study are available from the corresponding author upon request.

## Supplementary Materials

Supplementary Materials Supplementary Figure 1: sequence of BAC clones for DNA probe preparation. The name of the BAC clone is depicted in green, and the name of the gene in red. Supplementary Figure 2: inhibition of RNAP II is time-dependent. Supplementary Figure 3: sections of one single nucleus of an hPSC in 3D visualization of long-range interaction between POU5F1 (green) and CDYL (red) gene loci (both located on chromosome 6) show sequential appearance of signals within the nucleus. Supplementary Figure 4: distance distribution between POU5F1 and the selected genes in hPSCs and early differentiated cells and upon RNAP II inhibition. Supplementary Figure 5: distance distribution between individual alleles of the selected gene loci in hPSCs and early differentiated cells and upon RNAP II inhibition. Supplementary Table 1: long-range interactions (distance ≤ 2 *μ*m) in % between POU5F1 and the selected gene loci in hPSCs and early differentiated cells and upon RNAP II inhibition. Supplementary Table 2: long-range interactions (distance ≤ 2 μm) in % between individual alleles of POU5F1 and the selected genes in hPSCs and early differentiated cells and upon RNAP II inhibition.

## References

[1] Thomson J. A., Itskovitz-Eldor J., Shapiro S. S. (1998). Embryonic stem cell lines derived from human blastocysts. *Science*.

[2] Takahashi K., Tanabe K., Ohnuki M. (2007). Induction of pluripotent stem cells from adult human fibroblasts by defined factors. *Cell*.

[3] Meshorer E., Misteli T. (2006). Chromatin in pluripotent embryonic stem cells and differentiation. *Nature Reviews Molecular Cell Biology*.

[4] Bártová E., Krejčí J., Harničarová A., Kozubek S. (2008). Differentiation of human embryonic stem cells induces condensation of chromosome territories and formation of heterochromatin protein 1 foci. *Differentiation*.

[5] Rao S. S., Huntley M. H., Durand N. C. (2014). A 3D map of the human genome at kilobase resolution reveals principles of chromatin looping. *Cell*.

[6] Bouwman B. A. M., de Laat W. (2015). Architectural hallmarks of the pluripotent genome. *FEBS Letters*.

[7] Neph S., Stergachis A. B., Reynolds A., Sandstrom R., Borenstein E., Stamatoyannopoulos J. A. (2012). Circuitry and dynamics of human transcription factor regulatory networks. *Cell*.

[8] Jackson D. A., Hassan A. B., Errington R. J., Cook P. R. (1993). Visualization of focal sites of transcription within human nuclei. *The EMBO Journal*.

[9] Grande M. A., van der Kraan I., de Jong L., van Driel R. (1997). Nuclear distribution of transcription factors in relation to sites of transcription and RNA polymerase II. *Journal of Cell Science*.

[10] Verschure P. J., van der Kraan I., Manders E. M. M., van Driel R. (1999). Spatial relationship between transcription sites and chromosome territories. *The Journal of Cell Biology*.

[11] Olson M. O. J., Dundr M., Szebeni A. (2000). The nucleolus: an old factory with unexpected capabilities. *Trends in Cell Biology*.

[12] Jin D. J., Mata Martin C., Sun Z., Cagliero C., Zhou Y. N. (2016). Nucleolus-like compartmentalization of the transcription machinery in fast-growing bacterial cells. *Critical Reviews in Biochemistry and Molecular Biology*.

[13] Sanyal A., Lajoie B. R., Jain G., Dekker J. (2012). The long-range interaction landscape of gene promoters. *Nature*.

[14] Engel J. D., Tanimoto K. (2000). Looping, linking, and chromatin activity: new insights into beta-globin locus regulation. *Cell*.

[15] Liu Z., Garrard W. T. (2005). Long-range interactions between three transcriptional enhancers, active Vκ gene promoters, and a 3′ boundary sequence spanning 46 kilobases. *Molecular and Cellular Biology*.

[16] Ling J. Q., Li T., Hu J. F. (2006). CTCF mediates interchromosomal colocalization between Igf2/H19 and Wsb1/Nf1. *Science*.

[17] Apostolou E., Ferrari F., Walsh R. M. (2013). Genome-wide chromatin interactions of the Nanog locus in pluripotency, differentiation, and reprogramming. *Cell Stem Cell*.

[18] Jackson D. A., Iborra F. J., Manders E. M. M., Cook P. R. (1998). Numbers and organization of RNA polymerases, nascent transcripts, and transcription units in HeLa nuclei. *Molecular Biology of the Cell*.

[19] Osborne C. S. (2014). Molecular pathways: transcription factories and chromosomal translocations. *Clinical Cancer Research*.

[20] Creyghton M. P., Cheng A. W., Welstead G. G. (2010). Histone H3K27ac separates active from poised enhancers and predicts developmental state. *Proceedings of the National Academy of Sciences*.

[21] Ji X., Dadon D. B., Powell B. E. (2016). 3D chromosome regulatory landscape of human pluripotent cells. *Cell Stem Cell*.

[22] Bensaude O. (2011). Inhibiting eukaryotic transcription: which compound to choose? How to evaluate its activity?. *Transcription*.

[23] Sedlacek H. H. (2001). Mechanisms of action of flavopiridol. *Critical Reviews in Oncology/Hematology*.

[24] Baumli S., Lolli G., Lowe E. D. (2008). The structure of P-TEFb (CDK9/cyclin T1), its complex with flavopiridol and regulation by phosphorylation. *The EMBO Journal*.

[25] Wang L.-M., Ren D.-M. (2010). Flavopiridol, the first cyclin-dependent kinase inhibitor: recent advances in combination chemotherapy. *Mini Reviews in Medicinal Chemistry*.

[26] Abou-Nassar K., Brown J. (2010). Novel agents for the treatment of chronic lymphocytic leukemia. *Clinical Advances in Hematology & Oncology*.

[27] Niwa H., Miyazaki J., Smith A. G. (2000). Quantitative expression of Oct-3/4 defines differentiation, dedifferentiation or self-renewal of ES cells. *Nature Genetics*.

[28] Wei Z., Gao F., Kim S. (2013). Klf4 organizes long-range chromosomal interactions with the Oct4 locus in reprogramming and pluripotency. *Cell Stem Cell*.

[29] Gao F., Wei Z., An W., Wang K., Lu W. (2013). The interactomes of POU5F1 and SOX2 enhancers in human embryonic stem cells. *Scientific Reports*.

[30] Okuda T., Nishimura M., Nakao M., Fujitaa Y. (2001). RUNX1/AML1: a central player in hematopoiesis. *International Journal of Hematology*.

[31] Zhang J., Poh H. M., Peh S. Q. (2012). ChiA-PET analysis of transcriptional chromatin interactions. *Methods*.

[32] Initiative T. I., Adewumi O., Aflatoonian B. (2007). Characterization of human embryonic stem cell lines by the International Stem Cell Initiative. *Nature Biotechnology*.

[33] Simara P., Tesarova L., Rehakova D. (2018). Reprogramming of adult peripheral blood cells into human induced pluripotent stem cells as a safe and accessible source of endothelial cells. *Stem Cells and Development*.

[34] Svoboda D., Kozubek M., Stejskal S. (2009). Generation of digital phantoms of cell nuclei and simulation of image formation in 3D image cytometry. *Cytometry Part A*.

[35] Ulman V., Svoboda D., Nykter M., Kozubek M., Ruusuvuori P. (2016). Virtual cell imaging: a review on simulation methods employed in image cytometry. *Cytometry Part A*.

[36] Perlin K. (1985). An image synthesizer. *ACM SIGGRAPH Computer Graphics*.

[37] Svoboda D., Ulman V., Petrosino A. (2013). Towards a realistic distribution of cells in synthetically generated 3D cell populations. *Image Analysis and Processing – ICIAP 2013*.

[38] Lian X., Zhang J., Azarin S. M. (2013). Directed cardiomyocyte differentiation from human pluripotent stem cells by modulating Wnt/β-catenin signaling under fully defined conditions. *Nature Protocols*.

[39] Radzisheuskaya A., Le Bin Chia G., dos Santos R. L. (2013). A defined Oct4 level governs cell state transitions of pluripotency entry and differentiation into all embryonic lineages. *Nature Cell Biology*.

[40] Nichols J., Zevnik B., Anastassiadis K. (1998). Formation of pluripotent stem cells in the mammalian embryo depends on the POU transcription factor Oct4. *Cell*.

[41] Boyer L. A., Lee T. I., Cole M. F. (2005). Core transcriptional regulatory circuitry in human embryonic stem cells. *Cell*.

[42] Wang J., Rao S., Chu J. (2006). A protein interaction network for pluripotency of embryonic stem cells. *Nature*.

[43] Dean A. (2011). In the loop: long range chromatin interactions and gene regulation. *Briefings in Functional Genomics*.

[44] Dekker J., Marti-Renom M. A., Mirny L. A. (2013). Exploring the three-dimensional organization of genomes interpreting chromatin interaction data. *Nature Reviews Genetics*.

[45] Levsky J. M., Singer R. H. (2003). Fluorescence in situ hybridization: past, present and future. *Journal of Cell Science*.

[46] Joffe B., Leonhardt H., Solovei I. (2010). Differentiation and large scale spatial organization of the genome. *Current Opinion in Genetics and Development*.

[47] Solovei I., Cavallo A., Schermelleh L. (2002). Spatial preservation of nuclear chromatin architecture during three-dimensional fluorescence in situ hybridization (3D-FISH). *Experimental Cell Research*.

[48] Marsman J., Horsfield J. A. (2012). Long distance relationships: enhancer-promoter communication and dynamic gene transcription. *Biochimica et Biophysica Acta (BBA) - Gene Regulatory Mechanisms*.

[49] Augui S., Filion G. J., Huart S. (2007). Sensing X chromosome pairs before X inactivation via a novel X-pairing region of the Xic. *Science*.

[50] LaSalle J. M., Lalande M. (1996). Homologous association of oppositely imprinted chromosomal domains. *Science*.

[51] Masui O., Bonnet I., Le Baccon P. (2011). Live-cell chromosome dynamics and outcome of X chromosome pairing events during ES cell differentiation. *Cell*.

[52] Hogan M. S., Parfitt D.-E., Zepeda-Mendoza C. J., Shen M. M., Spector D. L. (2015). Transient pairing of homologous Oct4 alleles accompanies the onset of embryonic stem cell differentiation. *Cell Stem Cell*.

[53] Lieberman-Aiden E., van Berkum N. L., Williams L. (2009). Comprehensive mapping of long-range interactions reveals folding principles of the human genome. *Science*.

[54] Mattout A., Meshorer E. (2010). Chromatin plasticity and genome organization in pluripotent embryonic stem cells. *Current Opinion in Cell Biology*.

[55] Sood R., Kamikubo Y., Liu P. (2017). Role of RUNX1 in hematological malignancies. *Blood*.

[56] Escamilla-Del-Arenal M., da Rocha S. T., Spruijt C. G. (2013). Cdyl, a new partner of the inactive X chromosome and potential reader of H3K27me3 and H3K9me2. *Molecular and Cellular Biology*.

[57] Wan L., Hu X. J., Yan S. X. (2013). Generation and neuronal differentiation of induced pluripotent stem cells in Cdyl-/- mice. *Neuroreport*.

[58] Roth M., Bonev B., Lindsay J. (2010). FoxG1 and TLE2 act cooperatively to regulate ventral telencephalon formation. *Development*.

[59] Yu X., Zou J., Ye Z. (2008). Notch signaling activation in human embryonic stem cells is required for embryonic but not trophoblastic lineage commitment. *Cell Stem Cell*.

[60] Chen C.-Y., Liao W., Lou Y.-L. (2014). Inhibition of Notch signaling facilitates the differentiation of human-induced pluripotent stem cells into neural stem cells. *Molecular and Cellular Biochemistry*.

[61] Wang W., Yang J., Liu H. (2011). Rapid and efficient reprogramming of somatic cells to induced pluripotent stem cells by retinoic acid receptor gamma and liver receptor homolog 1. *Proceedings of the National Academy of Sciences*.

[62] Chatagnon A., Veber P., Morin V. (2015). RAR/RXR binding dynamics distinguish pluripotency from differentiation associated cis-regulatory elements. *Nucleic Acids Research*.

[63] Zeineddine D., Papadimou E., Chebli K. (2006). Oct-3/4 dose dependently regulates specification of embryonic stem cells toward a cardiac lineage and early heart development. *Developmental Cell*.

[64] Simandi Z., Horvath A., Wright L. C. (2016). OCT4 acts as an integrator of pluripotency and signal-induced differentiation. *Molecular Cell*.

