# Supplementary material for: The Role of RNA Polymerase II Contiguity and Long-Range Interactions in the Regulation of Gene Expression in Human Pluripotent Stem Cells
Source: Stem Cells Int. 2019 Feb 3;2019:1375807. doi: 10.1155/2019/1375807 (PMC6378007; doi:10.1155/2019/1375807)
Supplement: Supplementary Materials — Supplementary Figure 1: sequence of BAC clones for DNA probe preparation. The name of the BAC clone is depicted in green, and the name of the gene in red. Supplementary Figure 2: inhibition of RNAP II is time-dependent. Supplementary Figure 3: sections of one single nucleus of an hPSC in 3D visualization of long-range interaction between POU5F1 (green) and CDYL (red) gene loci (both located on chromosome 6) show sequential appearance of signals within the nucleus. Supplementary Figure 4: distance distribution between POU5F1 and the selected genes in hPSCs and early differentiated cells and upon RNAP II inhibition. Supplementary Figure 5: distance distribution between individual alleles of the selected gene loci in hPSCs and early differentiated cells and upon RNAP II inhibition. Supplementary Table 1: long-range interactions (distance ≤ 2 μm) in % between POU5F1 and the selected gene loci in hPSCs and early differentiated cells and upon RNAP II inhibition. Supplementary Table 2: long-range interactions (distance ≤ 2 μm) in % between individual alleles of POU5F1 and the selected genes in hPSCs and early differentiated cells and upon RNAP II inhibition. [file 1375807.f1.pdf]

Supplementary Materials

Supplementary Figure 1: Sequence of BAC clones for DNA probes preparation. Name of BAC clone is depicted in green, name of gene in red.

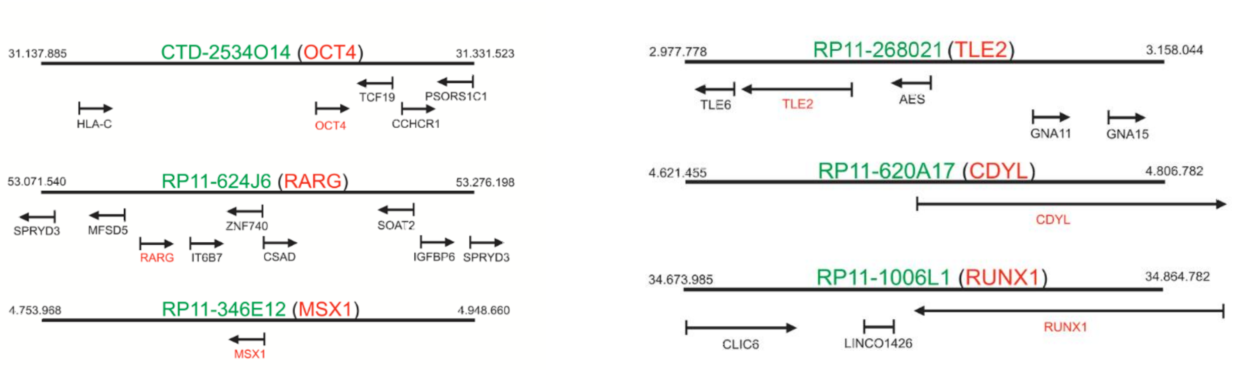

Suppl. Fig. 1: *Escherichia coli* carrying plasmids with sequences CTD-2534O14, RP11-624J6, RP11-346E12, RP11-268O21, RP11-1006L1 and RP11-620A17 were chosen from www.ncbi.nlm.nih.gov and acquired from Life Technologies BAC library. For BAC DNA isolation, see Materials and Methods section in the article.

Supplementary Figure 2: Inhibition of RNAP II is time-dependent.

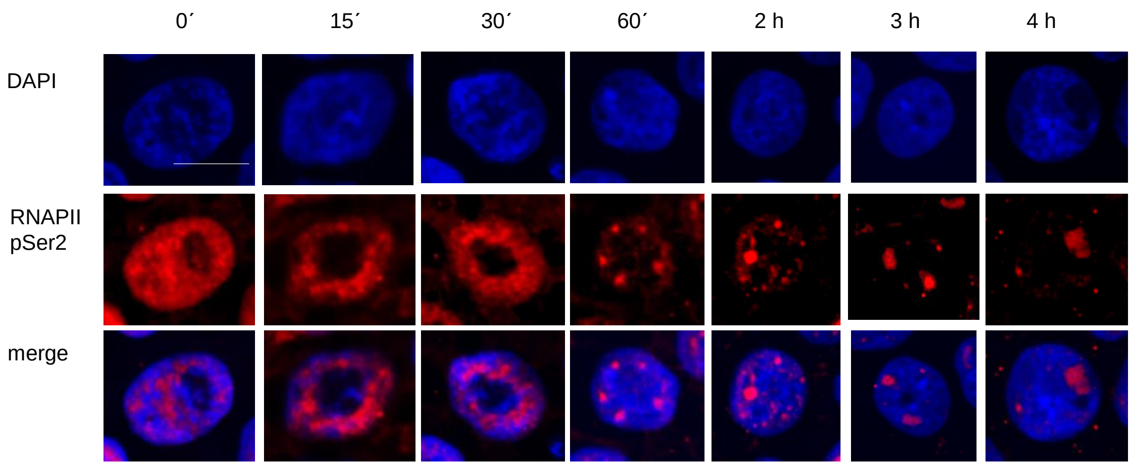

Suppl. Fig. 2: To study the effect of RNAP II inhibition on long-range interactions in hPSCs, we inhibited RNAP II using Flavopiridol that selectively pauses the transcription elongation phase. RNAP II in hPSCs was inhibited by 2 μM Flavopiridol for 15, 30, 60 min and 2, 3 and 4 hours to observe time-dependent manner of RNAP II inhibition. Cells were fixed with 4% paraformaldehyde (20 min, RT), permeabilized with 0.1% Triton-X100 in PBS (20 min, RT) and incubated with rabbit anti-RNAP II Ser2 primary antibody at 4 °C overnight. The next day, incubations with secondary antibodies conjugated to Alexa Fluor 594 were carried out at RT for 1 hour. Coverslips were mounted in DAPI-containing Mowiol. Microscopic analysis was performed using a Zeiss Axiovert 200M system. After RNAP II inhibition, transcription remains only in isolated regions within the nuclei. Scale bar: 10 μm.

Supplementary Figure 3: Sections of one single nucleus of hPSC in 3D visualization of long-range interaction between *POU5F1* (green) and *CDYL* (red) gene loci (both located on chromosome 6) show sequential appearance of signals within the nucleus.

a

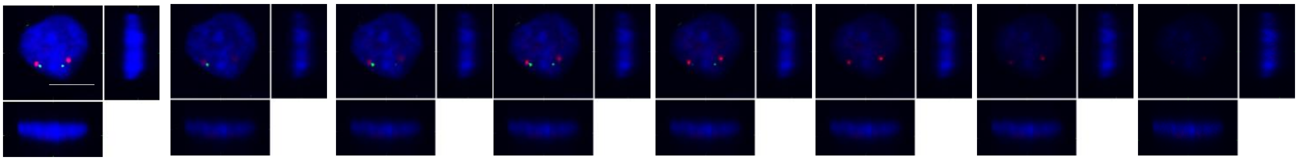

b

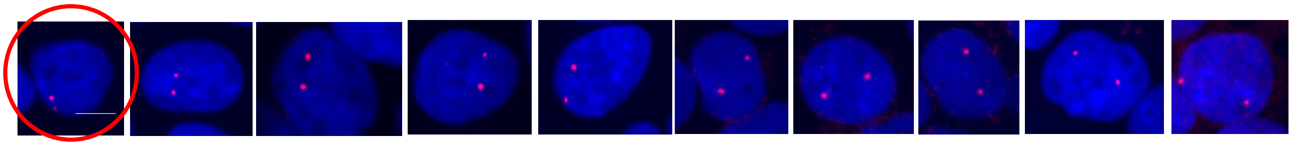

Suppl. Fig. 3:
a. The fractionalization of nuclei by 60 sections in the Z plane improved the visualization and positions of genes in 3D nuclei. Scale bar: 10 µm. For 3D FISH protocol, see Materials and Methods section in the article.
b. To reveal distance distribution, signals produced by 3D FISH (*POU5F1* gene loci in red) were separated in 14 distance intervals (µm) to cover appearance of selected genes in the nucleus. Only distance ≤ 2 µm (depicted in red circle) between two FISH signals was considerad as possible long-range interaction.

Supplementary Figure 4. Distance distribution between *POU5F1* and the selected genes in hPSCs, early differentiated cells and upon RNAP II inhibition.

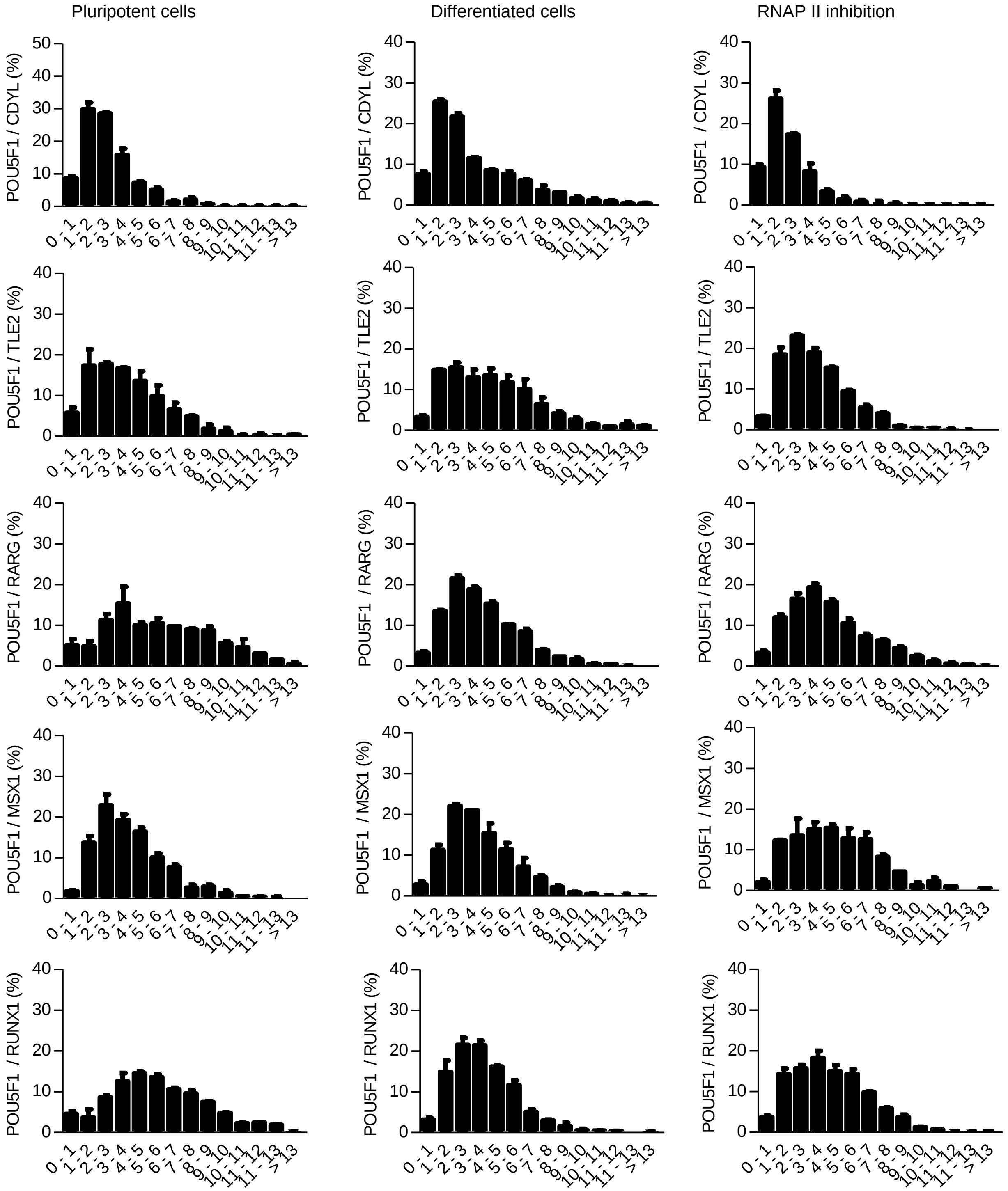

Suppl. Fig. 4: In order to reveal the distribution of signal spatial distances created by 3D-FISH throughout the nuclei of pluripotent cells and their differentiated counterparts and upon RNAP II inhibition, we separated signals produced by 3D-FISH in 14 intervals to cover the appearance of the selected genes in the nucleus. The vast majority of signals are located in relative proximity occupying the average distance of approximately 5 µm. The patterns of signal distributions of *POU5F1* and the selected genes tend to have a similar arrangement; the majority of signals from the selected genes lie in close proximity, implying the proximity of chromosomes. Mutual distance distribution (%) of FISH signals of *POU5F1* and selected gene loci in hPSCs, their differentiated counterparts and upon RNAP II inhibition display similar pattern and resemble normal distribution. For 3D FISH, at least 500 nuclei for each gene combination were counted, n=2. In charts columns show means, error bars show SEM.

Supplementary Figure 5. Distance distribution between individual alleles of selected gene loci in hPSCs, early differentiated cells and upon RNAP II inhibition.

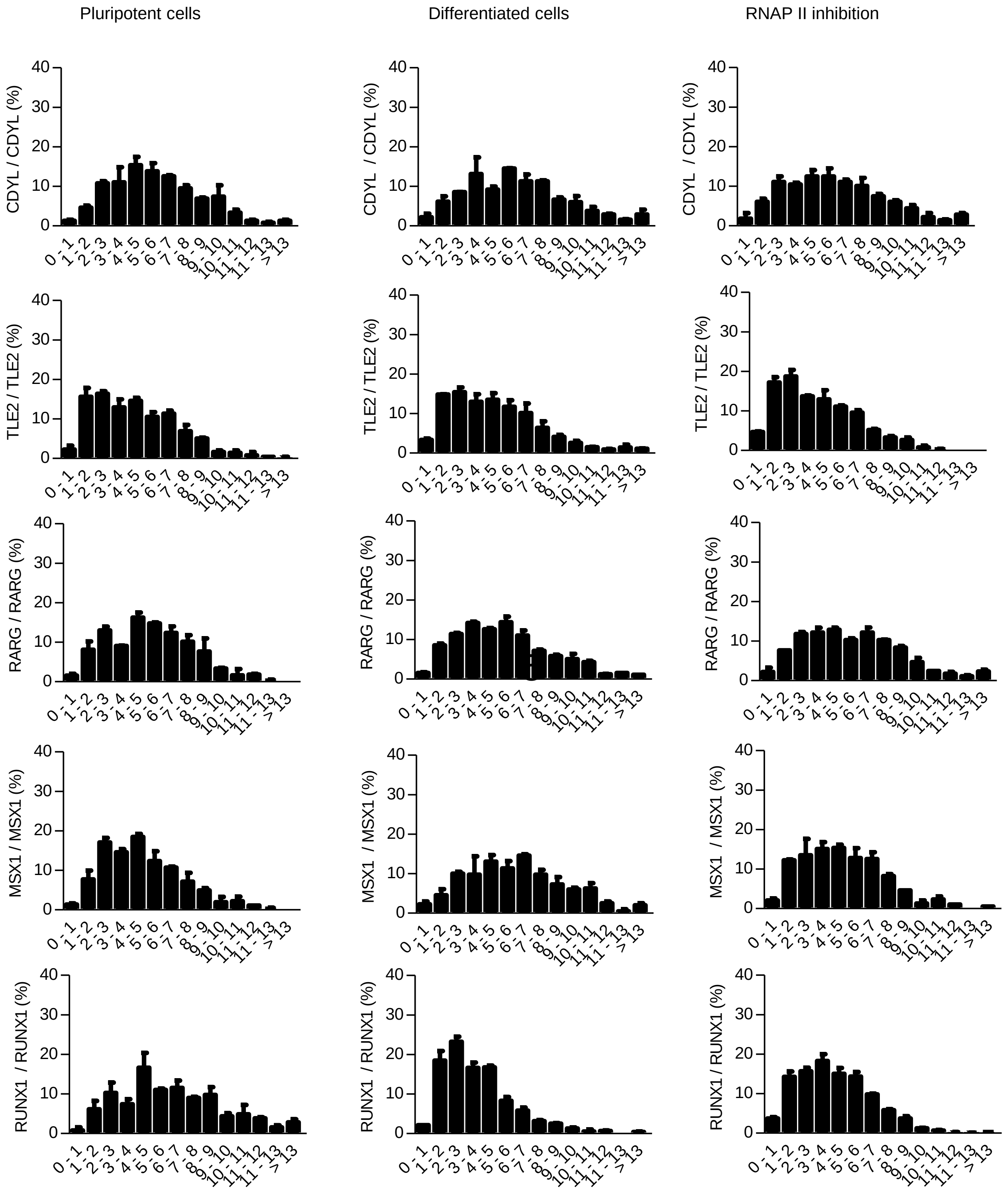

Suppl. Fig. 5: To cover distance distribution throughout the nuclei, we separated signals produced by 3D FISH in 14 distance intervals (μm) to cover appearance of selected genes in the nucleus. No significant changes compared with the distribution of the alleles of these genes throughout the nuclei. Mutual distance distribution (%) of individual alleles of followed genes produced by 3D FISH in hPSCs, their differentiated counterparts and upon RNAP II display similar pattern and resemble normal distribution. For 3D FISH, at least 500 nuclei for each gene combination were counted, n=2. In charts columns show means, error bars show SEM.

Supplementary Table 1: Long-range interactions (distance ≤ 2 µm) in % between *POU5F1* and the selected gene loci in hPSCs, early differentiated cells and upon RNAP II inhibition.

Gene combination	pluripotent cells (%)	differentiated cells (%)	RNAP II inhibition (%)
POU5F1/CDYL	38.45	33.11	35.6
POU5F1/TLE2	24.23	13.01	21.85
POU5F1/RARG	14.38	16.72	15.12
POU5F1/MSX1	15.56	14.07	15.64
POU5F1/RUNX1	8.22	18.1	11.85

Suppl. Table 1: We performed 3D-FISH analysis to visualize long-range interactions between *POU5F1* and genes *CDYL*, *TLE2*, *RARG*, *MSX1* and *RUNX1* in hPSCs, their differentiated counterparts and upon RNAP II inhibition. As a criterion for existing long-range interactions, we chose a distance lower or equal to 2 µm. For 3D FISH protocol, see Materials and Methods section in the article.

Supplementary Table 2: Long-range interactions (distance ≤ 2 µm) in % between individual alleles of *POU5F1* and the selected genes in hPSCs, early differentiated cells and upon RNAP II inhibition.

Gene combination	pluripotent cells (%)	differentiated cells (%)	RNAP II inhibition (%)
POU5F1/POU5F1	9.88	21.29	8.67
CDYL/CDYL	5.89	8.35	7.17
TLE2/TLE2	17.81	18.17	21.88
RARG/RARG	9.65	10.12	9.85
MSX1/MSX1	9.09	6.79	8.01
RUNX1/RUNX1	9.49	20.61	13.90

Suppl. Table 2: We performed 3D-FISH analysis to visualize long-range interactions between between individual alleles of genes *POU5F1*, *CDYL*, *TLE2*, *RARG*, *MSX1* and *RUNX1* in hPSCs, their differentiated counterparts and upon RNAP II inhibition. As a criterion for existing long-range interactions, we chose a distance lower or equal to 2 µm. For 3D FISH protocol, see Materials and Methods section in the article.
